# 2-(1*H*-Benzotriazol-1-yl)-1-(2-chloro­benzo­yl)ethyl 4-methyl­benzoate

**DOI:** 10.1107/S1600536808023222

**Published:** 2008-07-31

**Authors:** Lin-Bin Jiang, Lin Li, Yao Liu, Na-Na Tian, Jun Wan

**Affiliations:** a College of Chemistry and Chemical Engineering, Guangxi University, 530004 Nanning, Guangxi, People’s Republic of China; b College of Chemistry and Molecular Engineering, Qingdao University of Science and Technology, 266042 Qingdao, Shandong, People’s Republic of China

## Abstract

In the mol­ecule of the title compound, C_23_H_18_ClN_3_O_3_, the essentially planar benzotriazole ring makes dihedral angles of 52.93 (1) and 85.21 (1)°, respectively, with the chloro­phenyl and tolyl rings. The crystal packing is stabilized by π–π [centroid-to-centroid distance 3.830 (2) Å, interplanar distance 3.705 Å, slippage 0.968 Å]; C—H⋯π⋯tolyl ring inter­actions are also present.

## Related literature

For related literature, see: Bi *et al.*, (2007 ▶); Allen *et al.* (1987 ▶).
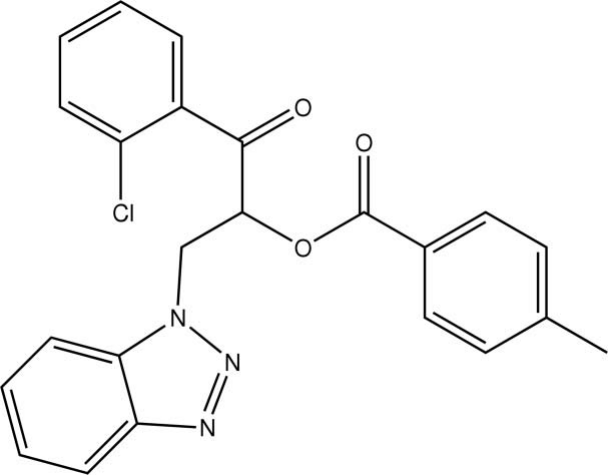


## Experimental

### 

#### Crystal data

C_23_H_18_ClN_3_O_3_
*M* *_r_* = 419.85
Monoclinic, 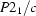

*a* = 7.9254 (7) Å
*b* = 26.151 (2) Å
*c* = 10.6002 (9) Å
β = 107.895 (1)°
*V* = 2090.7 (3) Å^3^
*Z* = 4
Mo *K*α radiation
μ = 0.21 mm^−1^
*T* = 293 (2) K
0.31 × 0.17 × 0.07 mm

#### Data collection

Siemens SMART 1000 CCD area-detector diffractometer
Absorption correction: multi-scan (*SADABS*; Sheldrick, 1996 ▶) *T*_min_ = 0.934, *T*_max_ = 0.983
11618 measured reflections
4112 independent reflections
2671 reflections with *I* > 2σ(*I*)
*R*_int_ = 0.032

#### Refinement

*R*[*F* ^2^ > 2σ(*F* ^2^)] = 0.058
*wR*(*F* ^2^) = 0.155
*S* = 1.02
4112 reflections
271 parameters
H-atom parameters constrained
Δρ_max_ = 0.35 e Å^−3^
Δρ_min_ = −0.14 e Å^−3^

### 

Data collection: *SMART* (Siemens, 1996 ▶); cell refinement: *SAINT* (Siemens, 1996 ▶); data reduction: *SAINT*; program(s) used to solve structure: *SHELXS97* (Sheldrick, 2008 ▶); program(s) used to refine structure: *SHELXL97* (Sheldrick, 2008 ▶); molecular graphics: *SHELXTL* (Sheldrick, 2008 ▶); software used to prepare material for publication: *SHELXTL*, *PARST* (Nardelli, 1995 ▶) and *PLATON* (Spek, 2003 ▶).

## Supplementary Material

Crystal structure: contains datablocks global, I. DOI: 10.1107/S1600536808023222/dn2368sup1.cif

Structure factors: contains datablocks I. DOI: 10.1107/S1600536808023222/dn2368Isup2.hkl

Additional supplementary materials:  crystallographic information; 3D view; checkCIF report

## Figures and Tables

**Table 1:** C—H⋯π interactions (Å, °) *Cg*4 is the centroid of the tolyl ring.

	C—H	C⋯*Cg*	C—H⋯*Cg*	H⋯*Cg*
C2—H2*B*⋯*Cg*4^ii^	0.93	3.879 (3)	168	2.96

Symmetry codes: (i) 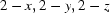; (ii) 
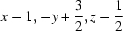
.

**Fig. 1.**
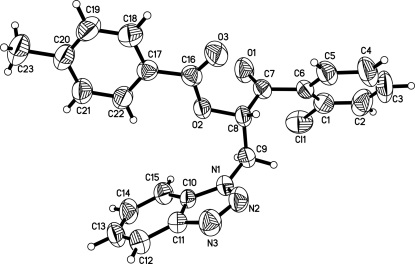
Molecular structure of compound (I) with the atom-labelling scheme. Ellipsoids are drawn at the 50% probability level. H atoms are represented as small spheres of arbitrary radii.

## supplementary crystallographic information

### Comment

Recently we have reported the structure of 2-(1*H*-benzotriazol-1-yl)-1-(2-fluorobenzoyl)ethyl benzoate (II) (Bi *et al.*, 2007). As part of our ongoing studies on benzotriazole derivatives with higher pharmacological activities, the title compound (I) was synthesized and its structure is shown here.

In (I), all bond lengths and angles are within normal ranges (Allen *et al.*, 1987) and are comparable with those in the related compound (II). In (I), the benzotriazole moiety is essentially planar with a dihedral angle of 0.46 (1)° between the N1–N3/C10/C11 triazole ring (A) and C10–C15 phenyl ring (B). The whole molecular is non-planar (Fig. 1). The benzotriazole system makes dihedral angles of 52.93 (1)° and 85.21 (1)° with the chlorophenyl C1–C6 (C) and the tolyl C17–C22 (D) rings respectively. The dihedral between the two phenyl rings, *viz*. C and D, is 34.40 (1)°.

The crystal packing is stabilized by slippest π–π and weak C—H···π interactions involving the tolyl ring D (Table 1).

### Experimental

The title compound was prepared according to the literature method of Bi *et al.* (2007). Single crystals suitable for X-ray diffraction were obtained by slow evaporation of an ethyl acetate solution at room temperature over a period of 6 d.

### Refinement

All H atoms were located in difference Fourier maps and constrained to ride on their parent atoms, with C—H distances in the range 0.93–0.97 Å, and with *U*_iso_(H) = 1.2 *U*_eq_(C) H atoms and 1.5 *U*_eq_(methyl C) H atoms.

### Crystal data

**Table:**

C_23_H_18_ClN_3_O_3_	*F*_000_ = 872
*M**_r_* = 419.85	*D*_x_ = 1.334 Mg m^−^^3^
Monoclinic, *P*2_1_/*c*	Mo *K*α radiation λ = 0.71073 Å
Hall symbol: -P 2ybc	Cell parameters from 2285 reflections
*a* = 7.9254 (7) Å	θ = 2.6–21.5º
*b* = 26.151 (2) Å	µ = 0.21 mm^−^^1^
*c* = 10.6002 (9) Å	*T* = 293 (2) K
β = 107.8950 (10)º	Plate, colourless
*V* = 2090.7 (3) Å^3^	0.31 × 0.17 × 0.07 mm
*Z* = 4	

### Data collection

**Table:**

Siemens SMART 1000 CCD area-detector diffractometer	4112 independent reflections
Radiation source: fine-focus sealed tube	2671 reflections with *I* > 2σ(*I*)
Monochromator: graphite	*R*_int_ = 0.032
Detector resolution: 8.33 pixels mm^-1^	θ_max_ = 26.0º
*T* = 293(2) K	θ_min_ = 2.2º
ω scans	*h* = −7→9
Absorption correction: multi-scan(SADABS; Sheldrick, 1996)	*k* = −31→32
*T*_min_ = 0.934, *T*_max_ = 0.983	*l* = −13→11
11618 measured reflections	

### Refinement

**Table:**

Refinement on *F*^2^	Secondary atom site location: difference Fourier map
Least-squares matrix: full	Hydrogen site location: inferred from neighbouring sites
*R*[*F*^2^ > 2σ(*F*^2^)] = 0.058	H-atom parameters constrained
*wR*(*F*^2^) = 0.155	*w* = 1/[σ^2^(*F*_o_^2^) + (0.0736*P*)^2^ + 0.3577*P*] where *P* = (*F*_o_^2^ + 2*F*_c_^2^)/3
*S* = 1.02	(Δ/σ)_max_ = 0.001
4112 reflections	Δρ_max_ = 0.35 e Å^−^^3^
271 parameters	Δρ_min_ = −0.14 e Å^−^^3^
Primary atom site location: structure-invariant direct methods	Extinction correction: none

### Special details

**Table:**

Geometry. All e.s.d.'s (except the e.s.d. in the dihedral angle between two l.s. planes) are estimated using the full covariance matrix. The cell e.s.d.'s are taken into account individually in the estimation of e.s.d.'s in distances, angles and torsion angles; correlations between e.s.d.'s in cell parameters are only used when they are defined by crystal symmetry. An approximate (isotropic) treatment of cell e.s.d.'s is used for estimating e.s.d.'s involving l.s. planes.
Refinement. Refinement of *F*^2^ against ALL reflections. The weighted *R*-factor *wR* and goodness of fit *S* are based on *F*^2^, conventional *R*-factors *R* are based on *F*, with *F* set to zero for negative *F*^2^. The threshold expression of *F*^2^ > σ(*F*^2^) is used only for calculating *R*-factors(gt) *etc*. and is not relevant to the choice of reflections for refinement. *R*-factors based on *F*^2^ are statistically about twice as large as those based on *F*, and *R*- factors based on ALL data will be even larger.

### Fractional atomic coordinates and isotropic or equivalent isotropic displacement parameters (Å^2^)

**Table:**

	*x*	*y*	*z*	*U*_iso_*/*U*_eq_	
Cl1	0.45657 (13)	0.67700 (3)	0.76685 (8)	0.0850 (3)	
O2	0.6915 (2)	0.85387 (6)	0.88074 (15)	0.0519 (4)	
N1	0.5321 (3)	0.85694 (8)	0.61201 (19)	0.0494 (5)	
C16	0.7125 (3)	0.88114 (9)	0.9930 (2)	0.0521 (6)	
O1	0.6508 (2)	0.76281 (7)	0.9880 (2)	0.0724 (6)	
C10	0.6819 (3)	0.87350 (9)	0.5879 (2)	0.0447 (6)	
C6	0.3402 (3)	0.75625 (9)	0.8833 (2)	0.0483 (6)	
O3	0.6005 (3)	0.88270 (8)	1.0475 (2)	0.0811 (6)	
C11	0.6356 (3)	0.92094 (10)	0.5281 (2)	0.0518 (6)	
N2	0.4019 (3)	0.89243 (9)	0.5702 (2)	0.0627 (6)	
C17	0.8850 (3)	0.90815 (9)	1.0357 (2)	0.0501 (6)	
N3	0.4614 (3)	0.93082 (9)	0.5193 (2)	0.0679 (6)	
C8	0.5253 (3)	0.82806 (9)	0.8302 (2)	0.0479 (6)	
H8A	0.4285	0.8511	0.8316	0.058*	
C22	1.0019 (3)	0.90722 (10)	0.9622 (3)	0.0606 (7)	
H22A	0.9731	0.8894	0.8825	0.073*	
C15	0.8502 (3)	0.85257 (11)	0.6109 (3)	0.0576 (7)	
H15A	0.8805	0.8207	0.6501	0.069*	
C7	0.5192 (3)	0.78034 (9)	0.9101 (2)	0.0493 (6)	
C9	0.5084 (3)	0.81338 (10)	0.6880 (2)	0.0537 (6)	
H9A	0.5967	0.7877	0.6881	0.064*	
H9B	0.3923	0.7986	0.6467	0.064*	
C20	1.2093 (4)	0.95912 (10)	1.1248 (3)	0.0649 (8)	
C5	0.2118 (4)	0.78141 (11)	0.9250 (3)	0.0644 (7)	
H5A	0.2369	0.8132	0.9659	0.077*	
C1	0.3004 (4)	0.70910 (10)	0.8229 (3)	0.0597 (7)	
C14	0.9684 (4)	0.88207 (12)	0.5720 (3)	0.0689 (8)	
H14A	1.0827	0.8698	0.5857	0.083*	
C2	0.1361 (5)	0.68742 (13)	0.8019 (3)	0.0889 (11)	
H2B	0.1093	0.6560	0.7591	0.107*	
C18	0.9309 (4)	0.93510 (11)	1.1532 (3)	0.0678 (8)	
H18A	0.8530	0.9365	1.2032	0.081*	
C12	0.7585 (4)	0.94992 (11)	0.4889 (3)	0.0681 (8)	
H12A	0.7290	0.9816	0.4483	0.082*	
C13	0.9234 (4)	0.92969 (12)	0.5129 (3)	0.0703 (8)	
H13A	1.0088	0.9483	0.4890	0.084*	
C19	1.0918 (4)	0.95997 (11)	1.1968 (3)	0.0745 (9)	
H19A	1.1214	0.9777	1.2766	0.089*	
C21	1.1613 (4)	0.93279 (11)	1.0071 (3)	0.0698 (8)	
H21A	1.2383	0.9322	0.9563	0.084*	
C23	1.3858 (4)	0.98627 (13)	1.1738 (3)	0.0903 (11)	
H23A	1.4513	0.9809	1.1124	0.135*	
H23B	1.4519	0.9730	1.2591	0.135*	
H23C	1.3663	1.0222	1.1812	0.135*	
C4	0.0476 (4)	0.75943 (16)	0.9057 (4)	0.0883 (11)	
H4B	−0.0379	0.7762	0.9338	0.106*	
C3	0.0112 (5)	0.71282 (17)	0.8449 (4)	0.1012 (13)	
H3B	−0.0994	0.6980	0.8325	0.121*	

### Atomic displacement parameters (Å^2^)

**Table:**

	*U*^11^	*U*^22^	*U*^33^	*U*^12^	*U*^13^	*U*^23^
Cl1	0.1093 (7)	0.0709 (5)	0.0759 (5)	0.0079 (4)	0.0300 (5)	−0.0074 (4)
O2	0.0451 (10)	0.0619 (11)	0.0536 (10)	−0.0130 (8)	0.0225 (8)	−0.0098 (8)
N1	0.0367 (11)	0.0624 (13)	0.0496 (11)	−0.0072 (10)	0.0142 (9)	0.0039 (10)
C16	0.0540 (16)	0.0540 (15)	0.0527 (15)	−0.0025 (12)	0.0228 (13)	−0.0039 (12)
O1	0.0438 (11)	0.0846 (14)	0.0856 (14)	0.0002 (10)	0.0152 (10)	0.0217 (11)
C10	0.0345 (13)	0.0591 (15)	0.0419 (12)	−0.0108 (11)	0.0137 (10)	−0.0052 (11)
C6	0.0427 (14)	0.0576 (15)	0.0465 (13)	−0.0047 (11)	0.0165 (11)	0.0085 (11)
O3	0.0696 (14)	0.1081 (16)	0.0804 (14)	−0.0179 (12)	0.0449 (12)	−0.0298 (12)
C11	0.0471 (16)	0.0581 (16)	0.0501 (14)	−0.0084 (12)	0.0149 (12)	−0.0014 (11)
N2	0.0390 (12)	0.0761 (16)	0.0724 (15)	−0.0028 (11)	0.0163 (11)	0.0049 (12)
C17	0.0545 (16)	0.0477 (13)	0.0482 (14)	−0.0009 (11)	0.0158 (12)	−0.0027 (11)
N3	0.0504 (15)	0.0699 (15)	0.0814 (16)	0.0030 (11)	0.0174 (12)	0.0120 (12)
C8	0.0378 (13)	0.0547 (15)	0.0548 (14)	−0.0090 (11)	0.0194 (11)	−0.0034 (11)
C22	0.0516 (16)	0.0700 (17)	0.0598 (16)	−0.0093 (13)	0.0164 (13)	−0.0141 (13)
C15	0.0457 (16)	0.0662 (17)	0.0640 (16)	−0.0009 (12)	0.0215 (13)	−0.0002 (13)
C7	0.0443 (15)	0.0570 (15)	0.0518 (14)	−0.0008 (12)	0.0225 (12)	−0.0018 (12)
C9	0.0494 (15)	0.0618 (16)	0.0532 (14)	−0.0150 (12)	0.0206 (12)	−0.0043 (12)
C20	0.0551 (17)	0.0583 (17)	0.0679 (18)	−0.0077 (13)	−0.0008 (14)	0.0008 (14)
C5	0.0508 (17)	0.0745 (18)	0.0741 (18)	0.0048 (14)	0.0283 (14)	0.0126 (15)
C1	0.0618 (18)	0.0566 (16)	0.0583 (16)	−0.0050 (13)	0.0150 (13)	0.0094 (13)
C14	0.0421 (15)	0.092 (2)	0.079 (2)	−0.0107 (15)	0.0283 (14)	−0.0150 (17)
C2	0.078 (3)	0.077 (2)	0.095 (2)	−0.0308 (19)	0.002 (2)	0.0156 (18)
C18	0.072 (2)	0.0725 (19)	0.0608 (17)	−0.0042 (15)	0.0226 (15)	−0.0104 (14)
C12	0.069 (2)	0.0677 (18)	0.0687 (18)	−0.0202 (15)	0.0221 (15)	0.0041 (14)
C13	0.064 (2)	0.085 (2)	0.0726 (19)	−0.0302 (17)	0.0367 (16)	−0.0098 (16)
C19	0.079 (2)	0.075 (2)	0.0608 (18)	−0.0149 (16)	0.0086 (16)	−0.0176 (15)
C21	0.0545 (17)	0.080 (2)	0.077 (2)	−0.0132 (15)	0.0236 (15)	−0.0113 (16)
C23	0.065 (2)	0.090 (2)	0.099 (2)	−0.0200 (17)	−0.0015 (17)	−0.0090 (19)
C4	0.0479 (19)	0.112 (3)	0.112 (3)	0.0068 (19)	0.0347 (18)	0.038 (2)
C3	0.048 (2)	0.116 (3)	0.130 (3)	−0.026 (2)	0.015 (2)	0.047 (3)

### Geometric parameters (Å, °)

**Table:**

Cl1—C1	1.744 (3)	C9—H9A	0.9700
O2—C16	1.353 (3)	C9—H9B	0.9700
O2—C8	1.429 (3)	C20—C21	1.372 (4)
N1—N2	1.357 (3)	C20—C19	1.374 (4)
N1—C10	1.360 (3)	C20—C23	1.512 (4)
N1—C9	1.441 (3)	C5—C4	1.379 (4)
C16—O3	1.200 (3)	C5—H5A	0.9300
C16—C17	1.480 (3)	C1—C2	1.374 (4)
O1—C7	1.204 (3)	C14—C13	1.391 (4)
C10—C11	1.390 (3)	C14—H14A	0.9300
C10—C15	1.392 (3)	C2—C3	1.381 (5)
C6—C1	1.381 (4)	C2—H2B	0.9300
C6—C5	1.392 (4)	C18—C19	1.378 (4)
C6—C7	1.498 (3)	C18—H18A	0.9300
C11—N3	1.380 (3)	C12—C13	1.359 (4)
C11—C12	1.394 (4)	C12—H12A	0.9300
N2—N3	1.296 (3)	C13—H13A	0.9300
C17—C18	1.379 (4)	C19—H19A	0.9300
C17—C22	1.382 (3)	C21—H21A	0.9300
C8—C7	1.517 (3)	C23—H23A	0.9600
C8—C9	1.521 (3)	C23—H23B	0.9600
C8—H8A	0.9800	C23—H23C	0.9600
C22—C21	1.378 (4)	C4—C3	1.367 (5)
C22—H22A	0.9300	C4—H4B	0.9300
C15—C14	1.371 (4)	C3—H3B	0.9300
C15—H15A	0.9300		
			
C16—O2—C8	115.37 (18)	C21—C20—C19	117.8 (3)
N2—N1—C10	109.96 (19)	C21—C20—C23	121.2 (3)
N2—N1—C9	120.5 (2)	C19—C20—C23	121.0 (3)
C10—N1—C9	128.9 (2)	C4—C5—C6	120.3 (3)
O3—C16—O2	122.2 (2)	C4—C5—H5A	119.9
O3—C16—C17	125.9 (2)	C6—C5—H5A	119.9
O2—C16—C17	111.9 (2)	C2—C1—C6	120.9 (3)
N1—C10—C11	104.3 (2)	C2—C1—Cl1	118.9 (3)
N1—C10—C15	133.4 (2)	C6—C1—Cl1	120.2 (2)
C11—C10—C15	122.3 (2)	C15—C14—C13	122.3 (3)
C1—C6—C5	119.0 (2)	C15—C14—H14A	118.8
C1—C6—C7	122.1 (2)	C13—C14—H14A	118.8
C5—C6—C7	118.9 (2)	C1—C2—C3	119.2 (3)
N3—C11—C10	108.4 (2)	C1—C2—H2B	120.4
N3—C11—C12	130.9 (3)	C3—C2—H2B	120.4
C10—C11—C12	120.7 (2)	C19—C18—C17	120.3 (3)
N3—N2—N1	109.2 (2)	C19—C18—H18A	119.9
C18—C17—C22	118.8 (3)	C17—C18—H18A	119.9
C18—C17—C16	118.9 (2)	C13—C12—C11	116.9 (3)
C22—C17—C16	122.3 (2)	C13—C12—H12A	121.6
N2—N3—C11	108.2 (2)	C11—C12—H12A	121.6
O2—C8—C7	111.28 (19)	C12—C13—C14	122.1 (3)
O2—C8—C9	106.32 (18)	C12—C13—H13A	118.9
C7—C8—C9	109.72 (19)	C14—C13—H13A	118.9
O2—C8—H8A	109.8	C20—C19—C18	121.4 (3)
C7—C8—H8A	109.8	C20—C19—H19A	119.3
C9—C8—H8A	109.8	C18—C19—H19A	119.3
C21—C22—C17	119.9 (3)	C20—C21—C22	121.8 (3)
C21—C22—H22A	120.0	C20—C21—H21A	119.1
C17—C22—H22A	120.0	C22—C21—H21A	119.1
C14—C15—C10	115.6 (3)	C20—C23—H23A	109.5
C14—C15—H15A	122.2	C20—C23—H23B	109.5
C10—C15—H15A	122.2	H23A—C23—H23B	109.5
O1—C7—C6	122.7 (2)	C20—C23—H23C	109.5
O1—C7—C8	121.8 (2)	H23A—C23—H23C	109.5
C6—C7—C8	115.5 (2)	H23B—C23—H23C	109.5
N1—C9—C8	111.6 (2)	C3—C4—C5	119.6 (3)
N1—C9—H9A	109.3	C3—C4—H4B	120.2
C8—C9—H9A	109.3	C5—C4—H4B	120.2
N1—C9—H9B	109.3	C4—C3—C2	121.0 (3)
C8—C9—H9B	109.3	C4—C3—H3B	119.5
H9A—C9—H9B	108.0	C2—C3—H3B	119.5
			
C8—O2—C16—O3	1.4 (3)	O2—C8—C7—C6	168.29 (19)
C8—O2—C16—C17	−177.87 (19)	C9—C8—C7—C6	−74.3 (3)
N2—N1—C10—C11	−0.5 (3)	N2—N1—C9—C8	−74.9 (3)
C9—N1—C10—C11	−170.9 (2)	C10—N1—C9—C8	94.6 (3)
N2—N1—C10—C15	179.8 (3)	O2—C8—C9—N1	−53.5 (3)
C9—N1—C10—C15	9.4 (4)	C7—C8—C9—N1	−174.0 (2)
N1—C10—C11—N3	0.2 (3)	C1—C6—C5—C4	−0.1 (4)
C15—C10—C11—N3	179.9 (2)	C7—C6—C5—C4	−177.9 (2)
N1—C10—C11—C12	179.9 (2)	C5—C6—C1—C2	1.0 (4)
C15—C10—C11—C12	−0.4 (4)	C7—C6—C1—C2	178.7 (2)
C10—N1—N2—N3	0.6 (3)	C5—C6—C1—Cl1	178.87 (19)
C9—N1—N2—N3	171.9 (2)	C7—C6—C1—Cl1	−3.4 (3)
O3—C16—C17—C18	4.8 (4)	C10—C15—C14—C13	−0.3 (4)
O2—C16—C17—C18	−176.0 (2)	C6—C1—C2—C3	−1.6 (5)
O3—C16—C17—C22	−175.4 (3)	Cl1—C1—C2—C3	−179.5 (2)
O2—C16—C17—C22	3.8 (3)	C22—C17—C18—C19	−0.9 (4)
N1—N2—N3—C11	−0.4 (3)	C16—C17—C18—C19	178.9 (2)
C10—C11—N3—N2	0.1 (3)	N3—C11—C12—C13	179.2 (3)
C12—C11—N3—N2	−179.5 (3)	C10—C11—C12—C13	−0.4 (4)
C16—O2—C8—C7	−76.5 (2)	C11—C12—C13—C14	0.8 (4)
C16—O2—C8—C9	164.1 (2)	C15—C14—C13—C12	−0.5 (5)
C18—C17—C22—C21	0.2 (4)	C21—C20—C19—C18	0.3 (4)
C16—C17—C22—C21	−179.6 (2)	C23—C20—C19—C18	−179.7 (3)
N1—C10—C15—C14	−179.6 (2)	C17—C18—C19—C20	0.6 (4)
C11—C10—C15—C14	0.7 (4)	C19—C20—C21—C22	−0.9 (4)
C1—C6—C7—O1	−66.6 (3)	C23—C20—C21—C22	179.0 (3)
C5—C6—C7—O1	111.1 (3)	C17—C22—C21—C20	0.7 (4)
C1—C6—C7—C8	112.1 (3)	C6—C5—C4—C3	−0.2 (5)
C5—C6—C7—C8	−70.2 (3)	C5—C4—C3—C2	−0.3 (5)
O2—C8—C7—O1	−12.9 (3)	C1—C2—C3—C4	1.2 (5)
C9—C8—C7—O1	104.5 (3)		

### Table 1 π–π and C—H···π interactions

Cg4 is the centroid of the tolyl ring. Symmetry codes: (i) 2-x, 2-y, 2-z; (ii) x-1, -y+3/2, z-1/2.

**Table:**

	Centroid–centroid (Å)	Interplanar distance (Å)	Slippage (Å)
Cg4···Cg4^i^	3.830 (2)	3.705	0.968
			
	C···Cg (Å)	C—H···Cg (°)	H···Cg (Å)
C2···Cg4^ii^	3.879 (3)	168	2.96
